# It's the Data Wot Won It: How Auditing a Waiting List in a London ADHD Team Reduced Waiting Times for Assessment

**DOI:** 10.1192/bjo.2026.11438

**Published:** 2026-06-30

**Authors:** Adam Montgomery

**Affiliations:** North East London NHS Foundation Trust, London, United Kingdom

## Abstract

**Aims:**

The ADHD service in the London Borough of Redbridge has a waiting list over nearly 600 patients. However, it was noticed that many patients were being booked in for assessments inappropriately, including patients who lived out of borough, who already had a diagnosis or who were seeking a medication review. It was decided to audit the waiting list to see if all remaining patients were suitable to be booked for assessment.

**Methods:**

The entire waiting list of 559 patients was screened manually and compared with our electronic patient record system Rio. Client demographics were checked to ensure they currently reside in Redbridge. Referral information was screened to ensure clients were referred for a diagnostic assessment. The list was also screened for duplicate patients. Theexpected standard was that 100% of patients on the waiting list were suitable for an ADHD assessment.

**Results:**

408/559 patients (73%) were deemed as suitable for assessment based on the criteria of living in Redbridge and requiring an ADHD assessment. 151 patients (27%) were unsuitable for a variety of reasons, including residing outside the borough, having moved since being referred, patients who were referred for medication review and patients who had not returned screening questionnaires. There were a significant number of duplicates, with 37 patients being on the list twice, and one patient three times. After removal of these 151 patients, the remaining 408 patients were reaudited to the same standard, with 398/408 (98%) of patients being suitable for assessment. The remaining 10 were subsequently removed. The removal of so many patients had the effect of reducing the waiting time for ADHD assessment in Redbridge by approximately 5–6 months.

**Conclusion:**

The audit revealed flaws with the waiting list and the screening system. Many patients were referred multiple times without this being noticed. There was no screening of the referral to assess which intervention the patient required (i.e. medication review, yearly review or new assessment), with all patients being added to the same list. Patient addresses were not scrutinised and there was no means of being alerted when patients had moved out of borough. These flaws resulted in an increased waiting time for ADHD assessment, and many patients being booked erroneously, wasting clinical and patient time. Measures to prevent this have been implemented, including cross-referencing of new referrals with the waiting list, regular screening of the waiting list by the consultant, and administrative staff checking patient addresses before offering appointments.

